# Cynaroside targets distinct metabolic pathways in gram-positive and gram-negative pathogenic bacteria: an integrated multi-omics study

**DOI:** 10.3389/fmicb.2026.1888286

**Published:** 2026-08-26

**Authors:** Shiqi Xia, Daofeng Liu, Haowen Zhang, Kui Zhao, Ming Jiang, Xiuhua Tao

**Affiliations:** 1 Jiangxi Academy of Forestry, Nanchang, Jiangxi, China; 2 Jiangxi Provincial Key Laboratory of Major Epidemics Prevention and Control, Jiangxi Provincial Center for Disease Control and Prevention, Nanchang, Jiangxi, China; 3 State Key Laboratory of Food Science and Resources, Nanchang University, Nanchang, Jiangxi, China

**Keywords:** antibacterial mechanism, cynaroside, gram-positive and gram-negative bacteria, metabolomics, transcriptomics, *Turpinia arguta*

## Abstract

The antibiotic resistance crisis has become a major threat to global public health. Discovering natural antibacterial compounds with unique mechanisms from traditional medicinal plants is an effective strategy to overcome this challenge. Through activity-guided fractionation, three compounds were isolated from *Turpinia arguta* leaves, identified as piperyamine A, cynaroside, and gallic acid. Cynaroside exhibited the strongest antibacterial activity, with MIC values of 31.25 μg/mL against *Staphylococcus aureus* (Gram-positive) and 62.5 μg/mL against *Vibrio parahaemolyticus* (Gram-negative). Phenotypic experiments, including scanning electron microscopy, electrical conductivity measurements, and alkaline phosphatase (ALP) activity assays, were performed to evaluate antibacterial effects of cynaroside. The results indicated that cynaroside exerted antibacterial effects by disrupting the integrity of bacterial cell walls and cell membranes, with markedly different responses between Gram-positive and Gram-negative bacteria. Specifically, the increase in electrical conductivity was more pronounced in *V. parahaemolyticus* (Gram-negative), while the peak ALP activity was higher in *S. aureus* (Gram-positive). Integrated metabolomic and transcriptomic analyses were conducted to elucidate the differential antibacterial mechanisms. In *S. aureus*, cynaroside treatment was associated with suppression of pyrimidine metabolism and histidine metabolism, negatively regulating 11 metabolites with *pyrC* as the hub gene; in *V. parahaemolyticus*, it mainly inhibited glyoxylate and dicarboxylate metabolism and branched-chain amino acid degradation, negatively regulating tricarboxylic acid cycle intermediates with *fdh3B* as the hub gene. This study reveals the differential antibacterial mechanisms of cynaroside isolated from *Turpinia arguta* against Gram-positive and Gram-negative bacteria, laying a theoretical foundation for the development of species-selective natural antibacterial agents.

## 1 Introduction

The antimicrobial resistance crisis is now recognized by the World Health Organization (WHO) as one of the top 10 global public health threats of the 21st century—a systemic risk with profound implications for modern medicine, food security, and economic stability (Rawson et al., 2025). Clinically, drug-resistant foodborne pathogens—such as *Staphylococcus aureus* (*S. aureus*) and *Vibrio parahaemolyticus* (*V. parahaemolyticus*)—pose increasing threats to both human and animal health, leading to higher morbidity, mortality, and treatment failure rates (Elbehiry et al., 2025). However, conventional antibiotics are increasingly ineffective against these resistant pathogens and often cause side effects such as gut microbiota disruption and host toxicity, highlighting the urgent need for alternative strategies (Peltak and Steen, 2025). Plant-derived natural compounds offer distinct advantages: they possess diverse chemical structures, exhibit multiple mechanisms of action that hinder resistance development, and generally have lower toxicity (Berteina-Raboin, 2025). Therefore, the discovery of natural antibacterial compounds from traditional medicinal plants has emerged as a promising approach to combat drug-resistant pathogens.

Among the vast array of medicinal plants with documented antimicrobial properties, the dried leaves of *Turpinia arguta* (Lindl.) Seem. (*T. arguta*) is a traditionally used medicinal herb in China with documented ethnopharmacological applications spanning centuries (Hu et al., 2024). The leaves have been legally included in the Chinese Pharmacopoeia since 2010 for the treatment of pharyngitis, tonsillitis, and tonsillar abscess (Ma et al., 2018). Pharmacological studies have demonstrated that the leaves possess antibacterial, anti-inflammatory, and analgesic effects (Ji et al., 2024). Our previous studies have demonstrated that extracts of *T. arguta* leaves exhibit potent antibacterial activity against both Gram-positive pathogens (including *S. aureus*) and Gram-negative pathogens (including *V. parahaemolyticus*). However, these studies have largely been confined to crude or semi-purified extracts, leaving the identity of the active constituents and their molecular mechanisms unclear.

Cynaroside (luteolin-7-O-*β*-D-glucoside), a flavonoid glycoside, has been reported to exhibit antibacterial activities against various pathogens, including *Staphylococcus aureus*, *Escherichia coli*, and *Pseudomonas aeruginosa* (Bouyahya et al., 2023). Recent studies have further demonstrated that cynaroside can suppress bacterial virulence and modulate host signaling pathways (Zheng et al., 2026). In addition, an MIC value of 100 μg/mL for cynaroside against *S. aureus* has been reported (Mogana et al., 2020). However, despite these extensive investigations, the molecular mechanisms underlying the antibacterial action of cynaroside—particularly its differential effects on Gram-positive versus Gram-negative bacteria—remain poorly understood. Most existing studies have evaluated its efficacy against individual strains, and no study has systematically compared how bacterial envelope architecture influences the metabolic and transcriptional responses to cynaroside treatment.

Piperyamine A, another compound isolated in this study, is a hydroxylamine-derived alkaloid that has been reported as a natural product from plant sources, although its antibacterial activity has been less extensively characterized compared to flavonoids. Gallic acid, the third compound identified in this study, is a well-known phenolic acid with documented antibacterial properties. Its antibacterial mechanisms have been attributed to the disruption of bacterial cell membranes, induction of oxidative stress, and inhibition of biofilm formation (Borges et al., 2013). However, similar to cynaroside, the species-specific mechanisms of these compounds—particularly their differential effects on Gram-positive versus Gram-negative bacteria—have not been systematically compared, leaving a significant gap in our understanding of their selective antibacterial action.

Beyond individual compounds, flavonoids as a class are recognized as multi-target antibacterial agents (Tan et al., 2022). However, the majority of existing studies evaluate antibacterial efficacy against individual reference strains, with no systematic comparative analysis of Gram-positive and Gram-negative pathogens. This gap is scientifically consequential: Gram-positive bacteria possess a thick, porous peptidoglycan layer embedded with teichoic acids, whereas Gram-negative species feature a highly selective outer membrane composed of lipopolysaccharide (LPS) (Halder and Mandal, 2018). Elucidating how secondary metabolites differentially interact with these distinct structural barriers is therefore essential for understanding their selective toxicity and for designing narrow-spectrum antimicrobials.

To address this gap, multi-omics technologies offer a powerful approach. Recent advances in multi-omics technologies have enabled systematic dissection of bacterial responses to antimicrobial stress at an unprecedented resolution (Nandi et al., 2025). Metabolomics can reveal the overall perturbation of bacterial metabolic networks at the metabolite level caused by compounds, while transcriptomics can analyze the regulatory mechanisms at the gene expression level. Integrating these two approaches allows construction of a “gene–metabolite–pathway” regulatory network to identify key metabolic nodes and hub genes (Wang et al., 2022), thereby systematically revealing the target sites and molecular mechanisms of antibacterial compounds. This strategy has been successfully applied in the research on the antibacterial mechanisms of various natural products (Liu et al., 2020; Zeng et al., 2024).

In this study, we isolated bioactive monomeric compounds from *T. arguta* leaves through activity-guided fractionation and identified their structures. Using scanning electron microscopy, membrane integrity assays, and integrated metabolomics and transcriptomics, we systematically investigated the differential antibacterial mechanisms of the most active compound against *S. aureus* (Gram-positive) and *V. parahaemolyticus* (Gram-negative). A gene–metabolite correlation network was constructed to identify key hub genes and metabolic nodes. Although cynaroside’s antibacterial activity has been previously reported against certain strains (Mogana et al., 2020), the species-specific molecular mechanisms underlying its differential effects on Gram-positive versus Gram-negative bacteria have not been systematically investigated. Here, we employ an integrated multi-omics approach to compare the metabolic and transcriptional responses of *S. aureus* and *V. parahaemolyticus* to cynaroside, revealing distinct pathway-level perturbations that reflect the structural and physiological differences between these two classes of bacteria. This work aims to provide a molecular basis for understanding the antibacterial activity of *T. arguta* leaves and to support the development of potential natural antibacterial agents with species-selective activity.

## 2 Materials and methods

### 2.1 Plant material and bacterial strains

The *T. arguta* leaves used in this study were collected in March 2024 from a cultivated population in Yifeng, Yichun City, Jiangxi Province, China (geographic coordinates: 28.45°N, 115.12°E). All plant materials were harvested from healthy *T. arguta* individuals grown under standardized horticultural conditions. Following collection, leaves were rinsed with distilled water to remove surface debris and soil residues, then air-dried in the shade until constant weight was achieved; dried samples were stored in opaque, airtight containers at 4 °C prior to extraction. Anhydrous ethanol, methanol, petroleum ether, ethyl acetate, and other chemical reagents were of analytical grade and purchased from Shanghai Aladdin Biochemical Technology Co., Ltd. (Shanghai, China).

*Staphylococcus aureus* CMCC(B)26003 and *V. parahaemolyticus* ATCC17802 were obtained from the Jiangxi Provincial Center for Disease Control and Prevention (Nanchang, China). Pure cultures were established by streaking onto solid agar media and incubating at 37 °C for 17 h. A single, well-isolated colony from each plate was transferred to liquid broth and grown under aerobic conditions at 37 °C with shaking at 150 rpm. Working suspensions were adjusted to a final concentration of (1.0 ± 0.1) × 10^6^ CFU/mL, as confirmed by viable counts, and used immediately for subsequent assays.

### 2.2 Extraction, isolation, and structural elucidation of antibacterial compounds

Activity-guided fractionation was employed to obtain the antibacterial constituents from *T. arguta* leaves, using the agar well diffusion assay (Kraiprom et al., 2026) against *S. aureus* and *V. parahaemolyticus* to monitor all separation steps.

**Figure 1:**
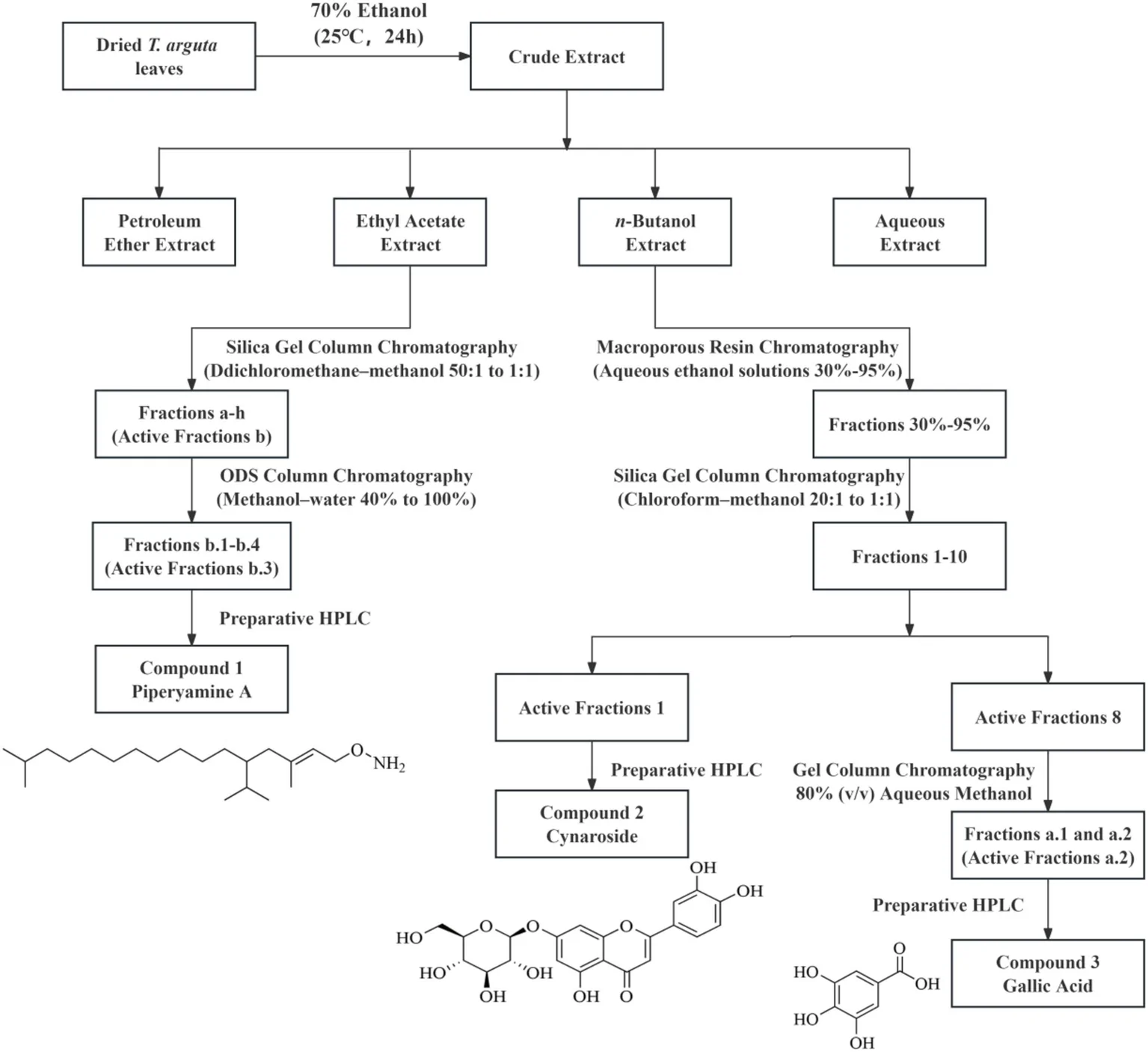
Schematic diagram of the isolation and purification process for compounds 1–3 from *T. arguta* leaves.

Dried *T. arguta* leaves (5.0 kg) were extracted three times with 70% ethanol at room temperature. The crude extract was sequentially partitioned with petroleum ether, ethyl acetate, *n*-butanol and water. The ethyl acetate and *n*-butanol fractions, which exhibited the strongest antibacterial activity (inhibition zones ≥ 8 mm), were subjected to repeated column chromatography (silica gel, reversed-phase octadecylsilyl, and macroporous resin) and preparative HPLC. This procedure yielded three monomeric compounds, designated as compounds 1, 2, and 3. The detailed isolation procedures are illustrated in Figure 1.

Structural elucidation of the isolated pure compounds was performed using ^1^H NMR and ^13^C NMR.

### 2.3 Determination of the minimum inhibitory concentration of three compounds

Following the method described by Andrews (2001) and Clinical and Laboratory Standards Institute (CLSI) guidelines, the MICs of the three isolated compounds against *S. aureus* and *V. parahaemolyticus* were determined using the standard broth microdilution method. Each compound was first dissolved in sterile deionized water containing 1% (v/v) dimethyl sulfoxide (DMSO) to prepare a stock solution of 1 mg/mL. The stock solutions were freshly prepared and sonicated for 5 min to ensure complete dissolution prior to each assay. Two-fold serial dilutions of each compound were prepared in 96-well microtiter plates, ranging from 1,000 to 0.98 μg/mL. Bacterial inocula were adjusted spectrophotometrically to approximately 1–2 × 10^8^ CFU/mL and then diluted to a final concentration of 5 × 10^5^ CFU/mL, as recommended by CLSI guidelines. Subsequently, 100 μL of the diluted bacterial suspension was added to each well containing 100 μL of serially diluted compound, yielding final compound concentrations ranging from 500 to 0.49 μg/mL. The plates were incubated at 37 °C for 18–20 h under aerobic conditions. The MIC was defined as the lowest concentration of each compound that completely inhibited visible bacterial growth, as determined by visual inspection and confirmed by optical density measurement at 600 nm (OD₆₀₀ < 0.1). Each well was examined in triplicate, and three independent biological replicates were performed for each compound against each bacterial strain.

Ampicillin (Sigma-Aldrich, USA) was included as a positive control in every independent experiment, and its MIC was determined under identical conditions to validate the assay system. The observed MIC values for ampicillin (2–4 μg/mL against both test strains) were consistent with CLSI reference ranges, confirming the reliability of the assay. Sterility controls and growth controls (bacteria without compound) were included on each plate to ensure the validity of the assay. The compound exhibiting the lowest MIC values against both *S. aureus* and *V. parahaemolyticus* was selected for subsequent mechanistic studies, including scanning electron microscopy, electrical conductivity measurement, alkaline phosphatase activity assay, metabolomics, and transcriptomics.

### 2.4 Analysis of scanning electron microscopy

Morphological alterations in *S. aureus* and *V. parahaemolyticus* following treatment with the cynaroside (compound 2) were assessed by scanning electron microscopy (SEM), following a modified protocol described previously (Liang et al., 2025). Briefly, 1 mL aliquots of each bacterial (*S. aureus* and *V. parahaemolyticus*) suspension (10^6^ CFU/mL) were harvested by centrifugation at 4,000 rpm for 8 min at 4 °C. Pellets were washed three times with sterile PBS (0.01 mol/L, pH 7.4) to remove residual culture medium. Bacteria were then resuspended in fresh sterile PBS containing the cynaroside at 1 × MIC (determined separately for each strain); vehicle controls received an equivalent volume of sterile PBS. Suspensions were incubated aerobically at 37 °C, and samples were collected at 0, 4, 8, and 12 h post-treatment. At each time point, cells were immediately pelleted by centrifugation at 4,000 rpm for 8 min at 4 °C, washed three times with sterile PBS, and fixed overnight at 4 °C in 2.5% (v/v) glutaraldehyde prepared in 0.1 M sodium cacodylate buffer (pH 7.4). Fixed samples were then dehydrated through a graded ethanol series (30, 50, 70, 80, and 95% v/v, each for 15 min at 4 °C), followed by two 15-min incubations in 100% ethanol. Dehydrated samples were then dried, sputter-coated with gold, and examined using a Hitachi S3000N SEM (Hitachi, Tokyo, Japan).

### 2.5 Determination of electrical conductivity

*Staphylococcus aureus* and *V. parahaemolyticus* (10^6^ CFU/mL) were centrifuged at 4,000 rpm for 8 min at 4 °C to pellet the cells. The supernatants were discarded, and the pellets were gently resuspended in sterile PBS (0.01 mol/L, pH 7.4) to remove residual medium components. For the experimental group, the cynaroside was added to achieve a final concentration of 1 × MIC; the control group received an equivalent volume of sterile PBS (vehicle control). Aliquots of both suspensions were incubated aerobically at 37 °C. At 0, 4, 8, and 12 h post-incubation, samples were centrifuged immediately at 4,000 rpm for 8 min at 4 °C, and the clarified supernatants were collected and stored on ice prior to electrical conductivity measurement using a calibrated conductivity meter (Shanghai INESA Scientific Instrument Co., Ltd., Shanghai, China).

### 2.6 Determination of alkaline phosphatase activity

Alkaline phosphatase (ALP) activity in *S. aureus* and *V. parahaemolyticus* was quantified spectrophotometrically using a commercially available ALP assay kit (Beyotime Biotechnology, Shanghai, China), with minor modifications. Briefly, bacterial suspensions (10^6^ CFU/mL) were centrifuged at 4,000 rpm for 8 min at 4 °C. The supernatants were discarded, and the pellets were gently resuspended in fresh sterile PBS (0.01 mol/L, pH 7.4) to remove residual culture components. The cynaroside (1 × MIC) was added to the experimental group; the control group received an equivalent volume of sterile PBS (vehicle control). Aliquots (1 mL) of each suspension were incubated aerobically at 37 °C. At 0, 4, 8, and 12 h post-incubation, samples were immediately withdrawn, centrifuged at 4,000 rpm for 8 min at 4 °C, and the clarified supernatants were collected and kept on ice. ALP activity was measured within 30 min of collection.

### 2.7 Metabolomic analysis

The cynaroside (compound 2) was added to *S. aureus* and *V. parahaemolyticus* suspension at 1 × MIC, and the cultures were incubated for 8 h at 37 °C, respectively. Following treatment, bacterial cells were harvested by centrifugation, rapidly frozen in liquid nitrogen, and stored at −80 °C until metabolite extraction. Non-targeted metabolomic profiling was performed using an LC–MS/MS platform at Majorbio Bio-Pharm Technology Co., Ltd. (Shanghai, China), with six biological replicates per group. Sample groups were designated as follows: SA-meta-C (*S. aureus* control, *n* = 6), SA-meta-T (*S. aureus* treated, *n* = 6), VP-meta-C (*V. parahaemolyticus* control, *n* = 6), and VP-meta-T (*V. parahaemolyticus* treated, *n* = 6). Unsupervised principal component analysis (PCA) and supervised orthogonal partial least squares discriminant analysis (OPLS-DA) were applied to identify statistically significant differential metabolites (DMs), with thresholds of variable importance in projection (VIP) > 1.0 and padj < 0.05. Significantly altered metabolites were mapped to the KEGG database for pathway enrichment analysis (*p* < 0.05), enabling systematic interpretation of the metabolic perturbations induced by the cynaroside.

### 2.8 Transcriptomic analysis

Bacterial culture and treatment conditions were identical to those used in the metabolomic profiling experiment. Bacterial cells were harvested by centrifugation, immediately flash-frozen in liquid nitrogen, and stored at −80 °C until RNA extraction, with three biological replicates per group. Sample groups were designated as follows: SA-tran-C (*S. aureus* control, *n* = 3), SA-tran-T (*S. aureus* treated, *n* = 3), VP-tran-C (*V. parahaemolyticus* control, *n* = 3), and VP-tran-T (*V. parahaemolyticus* treated, *n* = 3). Strand-specific cDNA libraries were constructed and sequenced on an Illumina NovaSeq 6,000 platform (paired-end, 150 bp). Differentially expressed genes (DEGs) were defined as those exhibiting |log₂(Fold Change)| ≥ 1 and padj < 0.05.

### 2.9 Integrated transcriptomic and metabolomic analysis

To systematically decipher the antibacterial mechanism of the cynaroside, integrative transcriptome and metabolome analysis was conducted. DEGs and their significantly enriched KEGG pathways were cross-mapped with DMs (VIP > 1.0, padj < 0.05) and their perturbed KEGG pathways. This hierarchical integration enabled the construction of a “gene–metabolite–pathway” regulatory network, revealing key metabolic nodes and coordinated transcriptional–metabolic responses underlying the antibacterial activity of the cynaroside.

### 2.10 Quantitative real-time PCR (qRT-PCR) validation

To validate the RNA-seq results, six selected differentially expressed genes were analyzed by qRT-PCR using the same RNA samples as those used for RNA-seq. Three independent biological replicates were included for each group. Species-specific bacterial housekeeping genes were used for normalization: *gyrB* for *S. aureus* and *recA* for *V. parahaemolyticus*. The suitability of the reference genes was assessed by examining the stability of their Ct values across the corresponding control and treatment groups. Relative gene expression was calculated using the 2^−ΔΔCt method (Wang et al., 2022) where ΔCt = Ct(target) − Ct(reference), and the mean ΔCt of the corresponding control group was used as the calibrator. Primer sequences are provided in Supplementary Table S1.

## 3 Results

### 3.1 Structural identification of the three compounds

Three compounds were isolated from *T. arguta* leaves. Based on NMR analysis, compound 3 was identified as gallic acid (Wang et al., 2024), and compound 1 as a hydroxylamine derivative named piperyamine A, which is reported here for the first time from this species (Siswina et al., 2023). Compound 2 was identified as a luteolin glucoside, and its NMR data were consistent with those previously reported for cynaroside (luteolin-7-O-*β*-D-glucoside) (Odontuya et al., 2005). All original spectra and detailed structural assignments are provided in the Supplementary Figures S1–S6 and Supplementary Tables S2–S4.

### 3.2 Antibacterial activity of the three compounds

The antibacterial activities of the three compounds were evaluated by MIC assay, and the results are summarized in (Supplementary Table S5). Among the three isolates, cynaroside (compound 2) exhibited the most potent antibacterial activity, with MIC values of 31.25 μg/mL against *S. aureus* and 62.5 μg/mL against *V. parahaemolyticus*. Piperyamine A (compound 1) showed moderate activity, with MIC values of 500 μg/mL against both tested strains. Gallic acid (compound 3) displayed relatively weak activity, with MIC values of 62.5 μg/mL against *S. aureus* and 125 μg/mL against *V. parahaemolyticus*. Under the same experimental conditions, the positive control ampicillin exhibited MIC values of 2 μg/mL against *S. aureus* and 4 μg/mL against *V. parahaemolyticus*.

### 3.3 Effects of cynaroside on bacterial morphology and cell-envelope integrity

**Figure 2:**
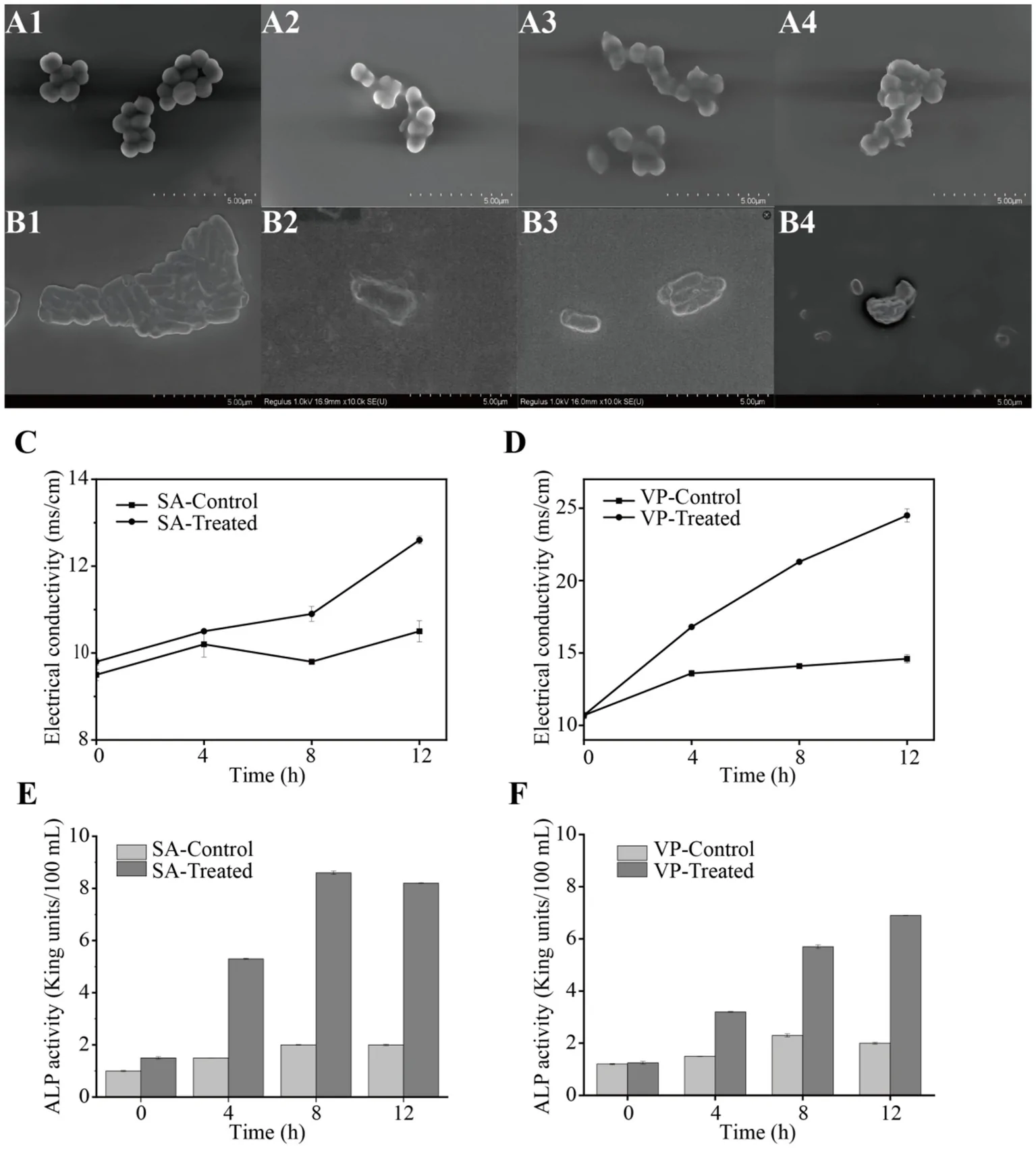
Effects of cynaroside on the morphology and cell envelope integrity of *S. aureus* and *V. parahaemolyticus*. **(A1–A4)** Scanning electron microscopy (SEM) images of *S. aureus* treated with cynaroside at 1× MIC for 0, 4, 8, and 12 h (left to right). Scale bar = 5 μm. **(B1–B4)** SEM images of *V. parahaemolyticus* treated with cynaroside at 1× MIC for 0, 4, 8, and 12 h (left to right). Scale bar = 5 μm. **(C, D)** Time-dependent changes in the electrical conductivity of *S. aureus* **(C)** and *V. parahaemolyticus* **(D)** suspensions after treatment with cynaroside (1× MIC). **(E,F)** Time-dependent changes in extracellular alkaline phosphatase (ALP) activity of *S. aureus* **(E)** and *V. parahaemolyticus* **(F)** after treatment with cynaroside (1× MIC). Data are presented as mean ± SD (*n* = 3 independent experiments).

The morphological changes in bacterial cells following treatment with cynaroside were examined by scanning electron microscopy (SEM). Untreated *S. aureus* cells displayed a typical spherical shape with smooth surfaces and clearly defined boundaries (Figure 2A1). In contrast, cynaroside-treated cells exhibited marked surface alterations, including protrusions, invaginations, surface collapse, and irregular or disrupted cellular margins (Figures 2A2–4). Similarly, untreated *V. parahaemolyticus* cells appeared as uniform, curved rods with intact structures (Figure 2B1), whereas treated cells showed pronounced deformation, indentation, surface disruption, aggregation, and adhesion (Figures 2B2–4). These observations demonstrate treatment-associated morphological damage but do not, by themselves, establish whether cell-envelope disruption represents the initiating antibacterial event or a secondary consequence of intracellular dysfunction.

The effects of the cynaroside on cell membranes were further evaluated by measuring the electrical conductivity of bacterial suspensions and the extracellular activity of alkaline phosphatase (ALP). Over the course of treatment, the electrical conductivity of the bacterial suspension in the untreated control group remained essentially unchanged. In contrast, exposure to cynaroside induced a time-dependent increase in electrical conductivity for both bacterial strains (Figures 2C,D). Notably, under identical treatment conditions, the magnitude of electrical conductivity increase was marginally greater for *V. parahaemolyticus* than for *S. aureus*.

Alkaline phosphatase is a cell-envelope-associated enzyme. In Gram-negative bacteria, it is localized in the periplasmic space between the inner and outer membranes; in Gram-positive bacteria, it is retained in the cell wall-membrane interface. Under normal physiological conditions, ALP cannot traverse an intact cell envelope. Consequently, the detection of ALP activity in the extracellular medium was interpreted as an indicator of compromised cell envelope integrity. In the treatment groups, ALP activity increased progressively for both strains. For *S. aureus*, the highest measured ALP activity was observed at 8–12 h after treatment, reaching approximately 8–10 King units/100 mL (Figure 2E). In contrast, *V. parahaemolyticus* exhibited a sustained upward trend with a lower peak value (6–8 King units/100 mL) (Figure 2F).

### 3.4 Metabolomics analysis results

#### 3.4.1 Metabolite identification

To elucidate the antibacterial mechanism of cynaroside against *S. aureus* and *V. parahaemolyticus*, intracellular metabolite profiles were comparatively analyzed using LC–MS/MS under control (untreated) and treatment (exposed to 1× MIC of cynaroside) conditions. In *S. aureus*, 822 metabolites were detected in the SA-meta-C group and 683 in the SA-meta-T group, with 540 metabolites overlapping between the two groups (Supplementary Figure S7A). After rigorous data preprocessing—including peak alignment, noise filtering, and retention time correction—and subsequent annotation via tandem mass spectrometry (MS/MS) fragmentation patterns matched against reference spectra, 287 metabolites were confidently identified and mapped to the Human Metabolome Database (HMDB), covering 11 chemical superclasses. The three most represented superclasses were Organic acids and derivatives (*n* = 90), Organoheterocyclic compounds (*n* = 54), and Phenylpropanoids and polyketides (*n* = 36) (Supplementary Figure S7C). In *V. parahaemolyticus*, 2,187 metabolites were detected in the VP-meta-C group and 1,314 in the VP-meta-T group, with 1,202 shared metabolites (Supplementary Figure S7B). Following identical analytical workflows, 628 metabolites were annotated in HMDB and classified into the same 11 superclasses; the top three were Organic acids and derivatives (*n* = 228), Lipids and lipid-like molecules (*n* = 130), and Organoheterocyclic compounds (*n* = 73) (Supplementary Figure S7D).

#### 3.4.2 Multivariate statistical analysis

**Figure 3:**
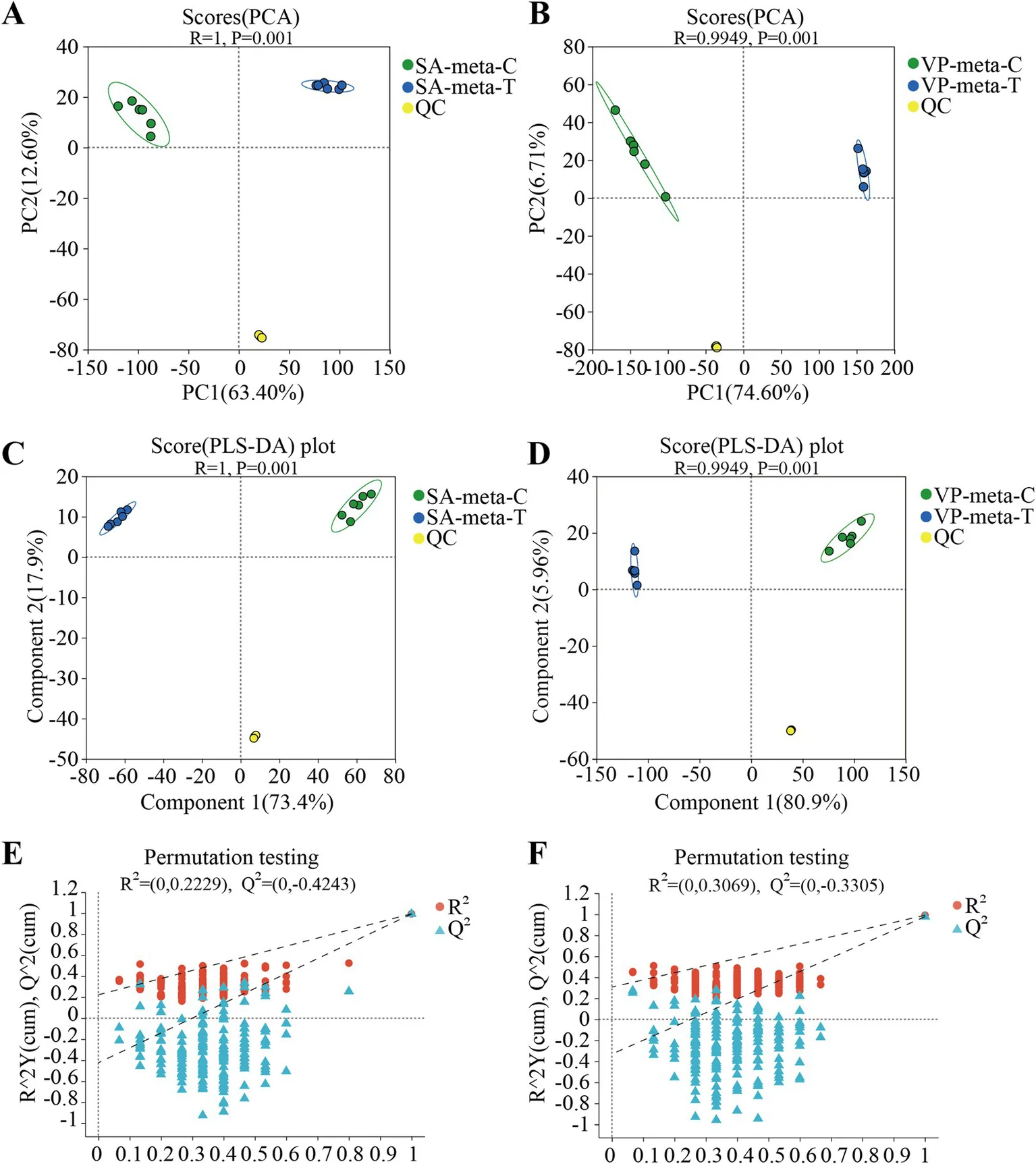
Multivariate statistical analysis of metabolomic data from *S. aureus* and *V. parahaemolyticus* following cynaroside treatment. **(A,B)** Principal component analysis (PCA) score plot of *S. aureus* **(A)** and *V. parahaemolyticus* **(B)** metabolomic data. Green circles: Control group; Blue squares: Cynaroside-treated group. **(C,D)** Orthogonal partial least squares discriminant analysis (OPLS-DA) score plot of *S. aureus* **(C)** and *V. parahaemolyticus* **(D)** metabolomic data. **(E,F)** Permutation test (200 permutations) for the OPLS-DA model of *S. aureus* **(E)** and *V. parahaemolyticus* **(F)**.

Principal component analysis (PCA) was performed to assess global metabolic profile alterations in *S. aureus* and *V. parahaemolyticus* following exposure to cynaroside. In *S. aureus*, the PCA score plot (Figure 3A) demonstrated distinct clustering between the SA-meta-C group and the SA-meta-T group, primarily driven by PC1, which accounted for 63.4% of the total variance; the cumulative contribution of PC1 and PC2 reached 76.0% (PC2: 12.6%). This pronounced separation indicates a substantial and systematic perturbation of the intracellular metabolome by cynaroside. To maximize discrimination power and isolate treatment-specific metabolic features, an orthogonal partial least squares discriminant analysis (OPLS-DA) model was built (Figure 3C). Model validity was rigorously evaluated using 200 response permutation tests. As shown in Figure 3E, the original model demonstrated high predictive ability with negative intercepts (*R*^2^ intercept = 0.2229, *Q*^2^ intercept = −0.4243), confirming model robustness and no overfitting.

#### 3.4.3 Differential metabolite analysis

In *V. parahaemolyticus*, PCA revealed even more pronounced group separation (Figure 3B), with PC1 alone explaining 74.6% of the variance and the first two components cumulatively accounting for 81.31% (PC2: 6.71%), reflecting a stronger and more coherent metabolic response to treatment. The OPLS-DA model (Figure 3D) showed clear class separation. Permutation testing (n=200; Figure 3F) validated the model with negative intercepts (*R*^2^ intercept = 0.3069, *Q*^2^ intercept = −0.3305), confirming stability and predictive accuracy for differential metabolite identification.

**Figure 4:**
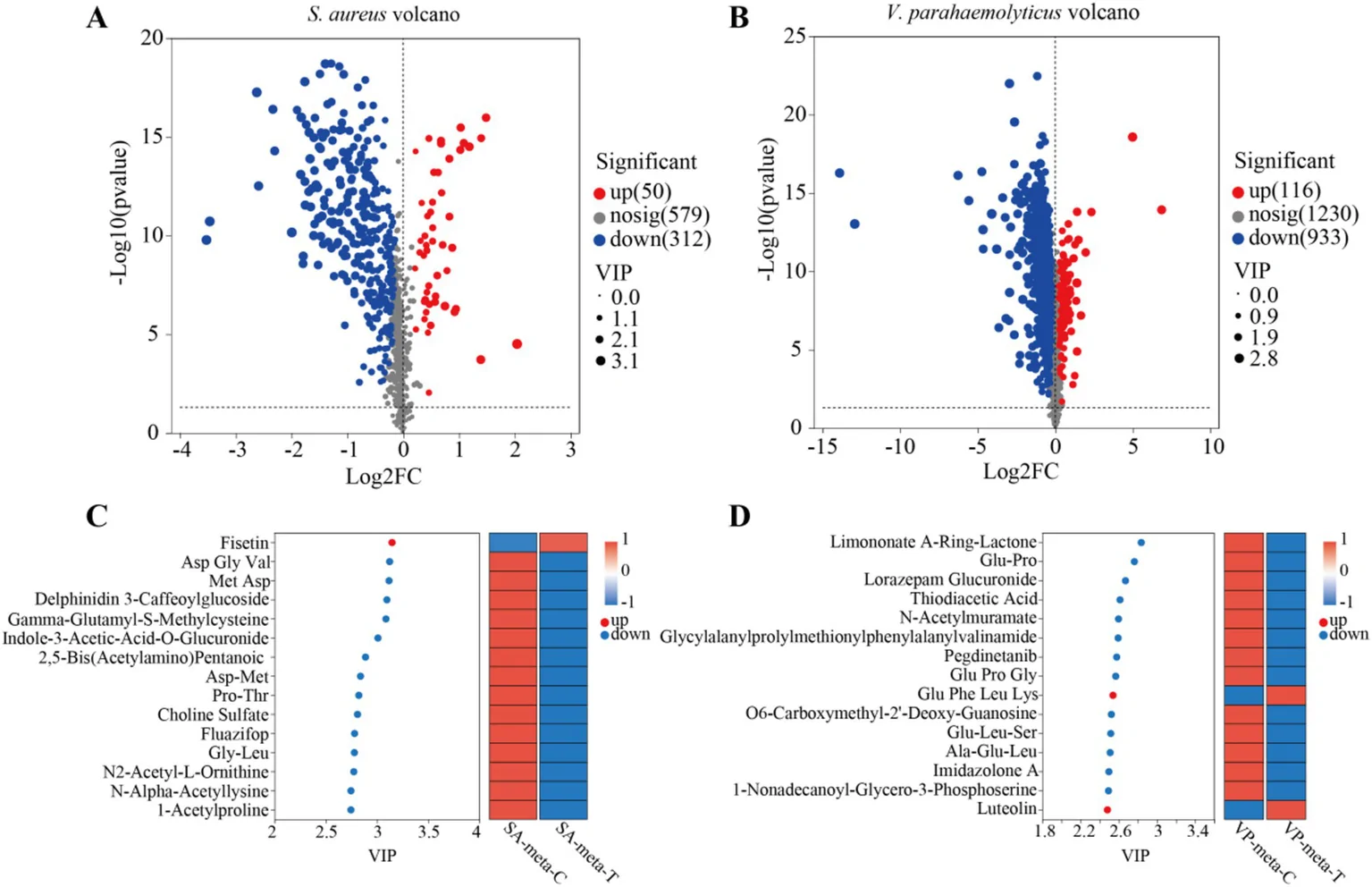
Differential metabolite analysis of *S. aureus* and *V. parahaemolyticus* following cynaroside treatment. **(A,B)** Volcano plot of differential metabolites in *S. aureus* **(A)** and *V. parahaemolyticus* **(B)**. **(C,D)** VIP score plot of the top 15 differential metabolites in *S. aureus* **(C)** and *V. parahaemolyticus* **(D)**. VIP values reflect the contribution of each metabolite to the OPLS-DA model; higher VIP values indicate greater discriminatory power.

Differential metabolites (DMs) were identified using the OPLS-DA model (VIP > 1, padj < 0.05). In *S. aureus*, 362 DMs (50 upregulated and 312 downregulated compounds) were detected (Figure 4A). In *V. parahaemolyticus*, 1,049 DMs identified—116 upregulated and 933 downregulated (Figure **4**B).

The top 15 metabolites ranked by VIP score were extracted from each OPLS-DA model. In *S. aureus*, these DMs exhibited VIP values ranging from 2.75 to 3.15 (Figure 4C); the highest-ranking DMs was fisetin (VIP = 3.15), followed by Asp-Gly-Val (VIP = 3.12) and Met-Asp (VIP = 3.11). In *V. parahaemolyticus*, the top 15 VIP DMs spanned 2.48–2.84 (Figure 4D); limonoate A-ring-lactone ranked first (VIP = 2.84), followed by Glu-Pro (VIP = 2.76) and lorazepam glucuronide (VIP = 2.67).

#### 3.4.4 Pathway enrichment analysis of differential metabolites

**Figure 5:**
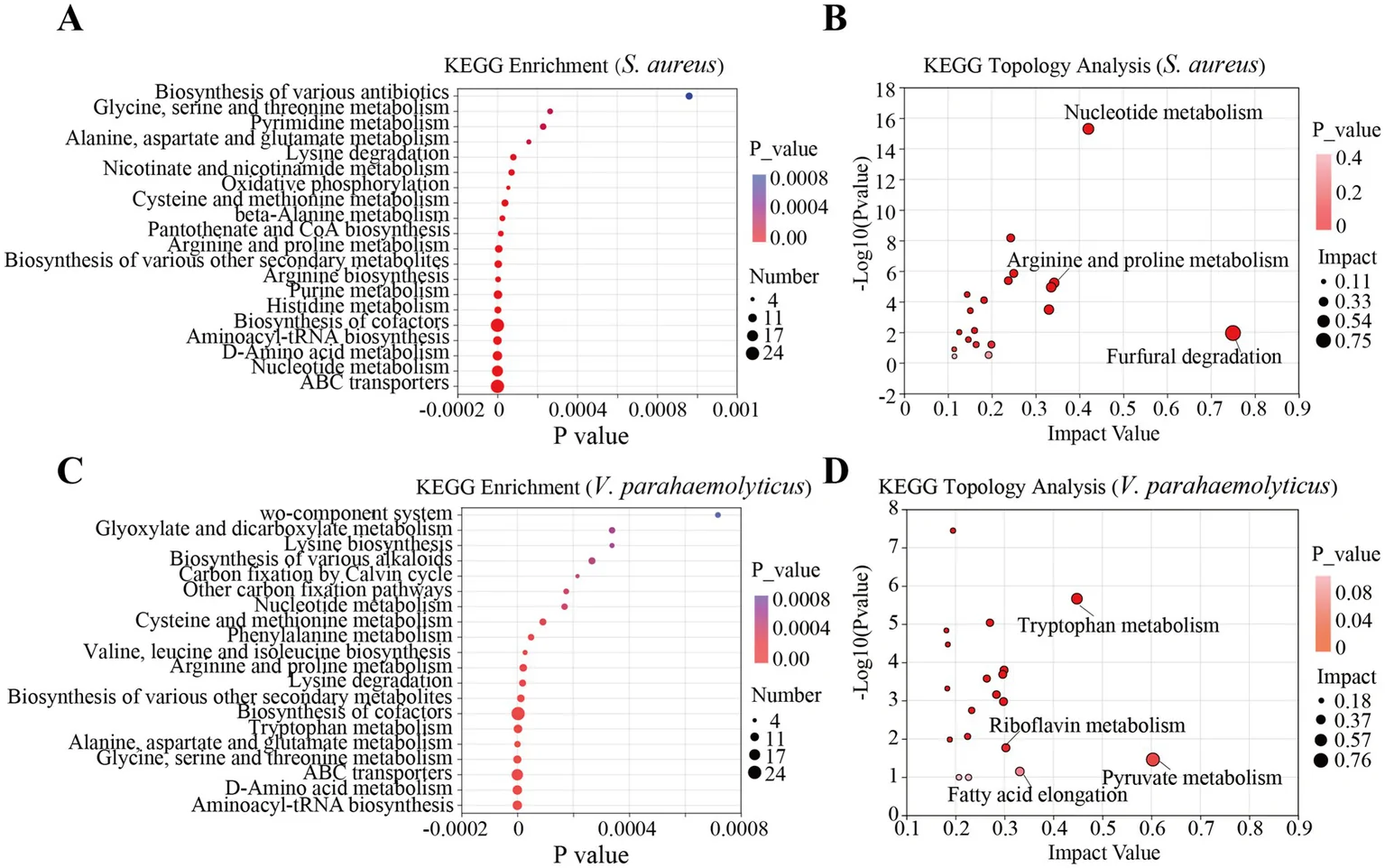
KEGG pathway enrichment analysis of differential metabolites in *S. aureus* and *V. parahaemolyticus* following cynaroside treatment. **(A,C)** Top 20 enriched KEGG pathways in *S. aureus* **(A)** and *V. parahaemolyticus* **(C)**. Significantly enriched pathways were identified using a threshold of *padj* < 0.05. The size of each bubble represents the number of metabolites mapped to the pathway; the color intensity reflects the significance level (darker colors indicate smaller *p*-values). **(B,D)** Topological pathway analysis of *S. aureus* **(B)** and *V. parahaemolyticus* **(D)** showing high-impact metabolites. The impact value reflects the relative importance of each metabolite within the pathway; metabolites with higher impact values are considered functional hubs.

KEGG pathway enrichment analysis identified the top 20 significantly enriched pathways in each bacterial strain. In *S. aureus*, the three most significantly enriched pathways were biosynthesis of various antibiotics, glycine, serine and threonine metabolism, and pyrimidine metabolism (Figure 5A). In *V. parahaemolyticus*, the top three were two-component system, glyoxylate and dicarboxylate metabolism, and lysine biosynthesis (Figure 5C).

Comparative analysis revealed that cynaroside exerts both conserved and species-specific metabolic perturbations. Conserved effects were observed in amino acid metabolism (alanine/aspartate/glutamate, cysteine/methionine, and lysine degradation pathways), nucleotide metabolism (purine and pyrimidine metabolism), genetic information processing (aminoacyl-tRNA biosynthesis), transport systems (ABC transporters), and secondary metabolism (biosynthesis of various secondary metabolites and antibiotics). Species-specific responses included: in *V. parahaemolyticus*, enrichment of the two-component system and suppression of lipopolysaccharide biosynthesis; in energy metabolism, *V. parahaemolyticus* showed comprehensive inhibition of carbon fixation pathways (other carbon fixation pathways, calvin cycle, glyoxylate and dicarboxylate metabolism), whereas *S. aureus* showed selective perturbation of oxidative phosphorylation.

Topological pathway analysis further prioritized high-impact metabolites. In *S. aureus*, key nodes included 2-Furoic acid in furfural degradation (Impact = 0.75); uracil and thymine in nucleotide metabolism (Impact = 0.42); and glutamic acid and spermine in arginine and proline metabolism (Impact = 0.34) (Figure 5B). In *V. parahaemolyticus*, high-impact nodes comprised pyruvic acid in pyruvate metabolism (Impact = 0.60); and L-Tryptophan and serotonin in tryptophan metabolism (Impact = 0.45) (Figure 5D). These metabolites serve as functional hubs whose dysregulation propagates cascading effects throughout their respective pathways, positioning them as high-priority candidates for mechanistic validation of cynaroside’s antibacterial action.

### 3.5 Transcriptomics analysis results

#### 3.5.1 RNA-Seq quality control and sample correlation analysis

To systematically investigate the transcriptional regulatory effects of cynaroside on *S. aureus* and *V. parahaemolyticus*, RNA sequencing was performed using the Illumina platform. Quality control metrics demonstrated high-fidelity sequencing: all samples achieved clean read Q30 scores exceeding 95.55% for *S. aureus* and 96.77% for *V. parahaemolyticus* (Supplementary Table S6). Clean reads were uniquely aligned to the respective reference genomes—*S. aureus* strain (RefSeq accession: GCF_000013425.1) and *V. parahaemolyticus* strain (RefSeq accession: GCF_000196095.1)—with average mapping rates of 94.3 and 94.5% (Supplementary Table S6), respectively. Principal component analysis (PCA) of normalized gene expression profiles revealed clear and reproducible separation between control and treatment groups in both strains (Supplementary Figure S8).

#### 3.5.2 Identification and functional enrichment analysis of differentially expressed genes

**Figure 6:**
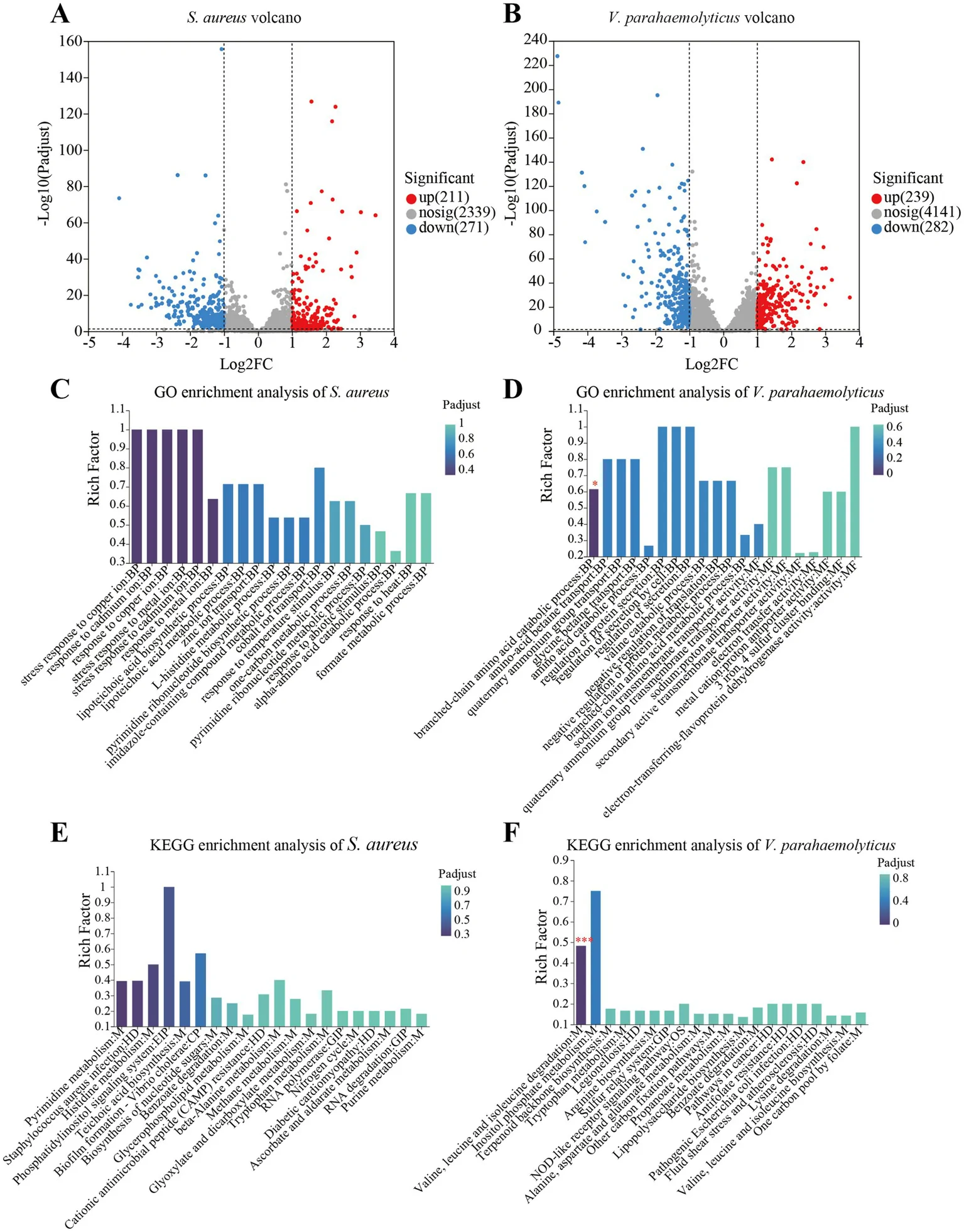
Transcriptomic analysis of *S. aureus* and *V. parahaemolyticus* following cynaroside treatment. **(A,B)** Volcano plots of differentially expressed genes (DEGs) in *S. aureus* **(A)** and *V. parahaemolyticus* **(B)** (*n* = 3 per group). DEGs were defined as genes with |*log₂*(fold change)| ≥ 1 and *padj* < 0.05. Red dots: Upregulated genes; blue dots: Downregulated genes; gray dots: Genes with no significant change. **(C,D)** GO enrichment analysis of *S. aureus* **(C)** and *V. parahaemolyticus* **(D)**, showing significantly enriched biological processes (*padj* < 0.05). The size of each bubble represents the number of genes enriched in the GO term. **(E,F)** KEGG pathway enrichment analysis of *S. aureus* **(E)** and *V. parahaemolyticus* **(F)**, showing significantly enriched pathways (*padj* < 0.05). The size of each bubble represents the number of DEGs mapped to the pathway.

In *S. aureus*, 492 DEGs were detected (221 upregulated, 271 downregulated); in *V. parahaemolyticus*, 521 DEGs were identified (239 upregulated, 282 downregulated) (Figures 6A,B).

GO enrichment analysis revealed both conserved and species-specific transcriptional perturbations (Figures 6C,D). Conserved across both strains were enrichments in amino acid metabolism (*S. aureus*: L-histidine metabolic process, *α*-amino acid catabolic process; *V. parahaemolyticus*: branched-chain amino acid catabolic process, valine catabolic process) and translation regulation (*S. aureus*: one-carbon metabolic process; *V. parahaemolyticus*: negative regulation of translation). Species-specific responses included: in *S. aureus*, stress response to metal ion and lipoteichoic acid biosynthesis; in *V. parahaemolyticus*, amino-acid betaine transport, regulation of secretion, and electron transfer activity.

KEGG pathway enrichment analysis further supported these findings (Figures 6E,F). Both strains showed perturbations in amino acid metabolism (*S. aureus*: histidine, beta-alanine, and tryptophan metabolism; *V. parahaemolyticus*: Valine, leucine, and isoleucine (BCAA) degradation and tryptophan metabolism) and energy metabolism (*S. aureus*: Glyoxylate and dicarboxylate metabolism, methane metabolism, nitrogen metabolism; *V. parahaemolyticus*: other carbon fixation pathways, propanoate metabolism, Calvin cycle). Species-specific pathways included: in *S. aureus*, lipid metabolism (phosphatidylinositol signaling, glycerophospholipid metabolism) and Gram-positive cell wall synthesis (teichoic acid biosynthesis, CAMP resistance); in *V. parahaemolyticus*, lipopolysaccharide biosynthesis, NOD-like receptor signaling, inositol phosphate metabolism, and terpenoid backbone biosynthesis.

### 3.6 Integrated metabolomics and transcriptomics analysis results

#### 3.6.1 Convergent pathway analysis

**Figure 7:**
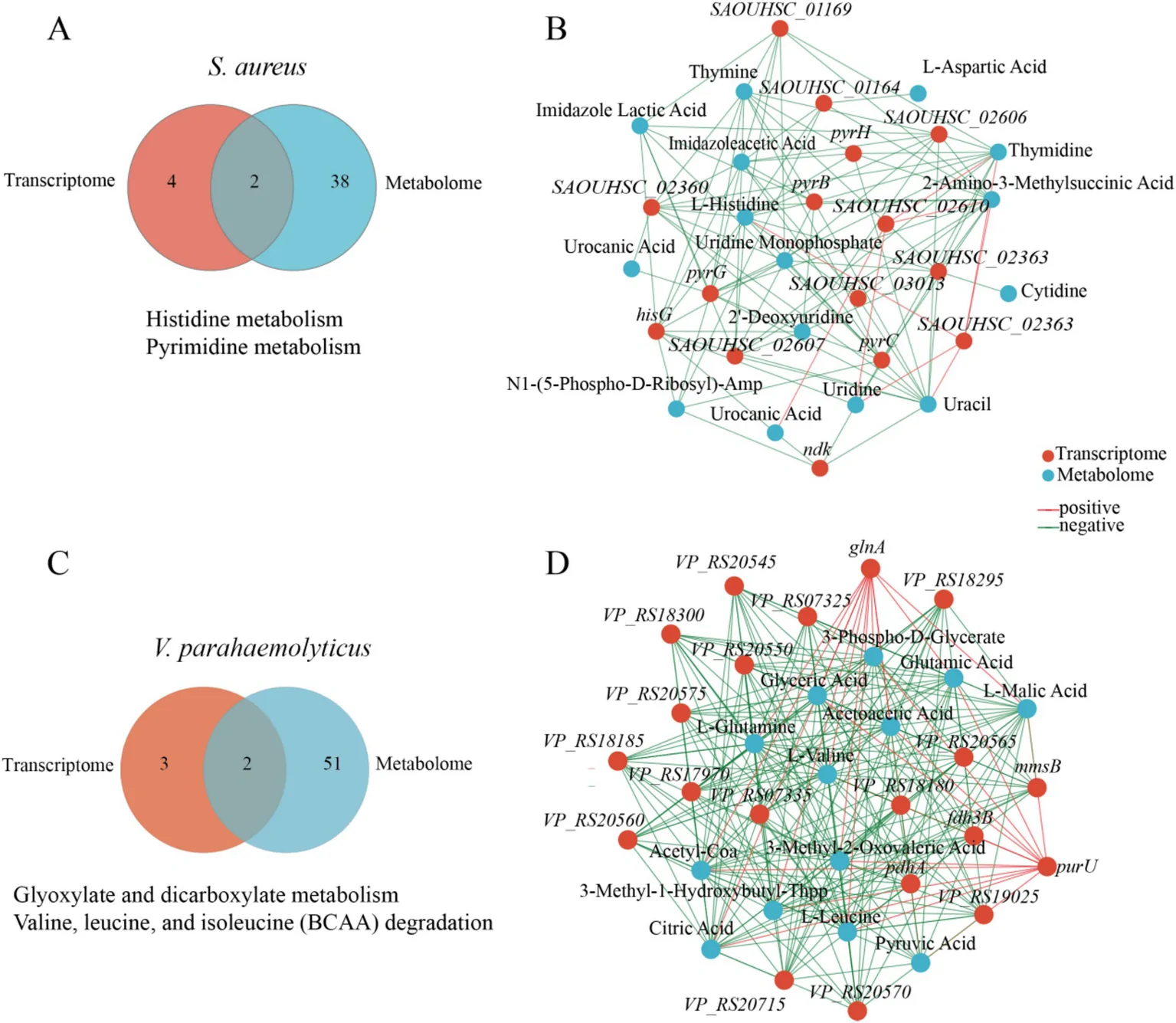
Integrated multi-omics analysis. **(A,C)** Shared KEGG pathways between metabolomics and transcriptomics in *S. aureus* **(A)** and *V. parahaemolyticus* **(C)**. Pathways shown are those significantly enriched (*padj* < 0.05) in both omics datasets. **(B,D)** Gene–metabolite correlation networks within convergent pathways in *S. aureus* **(B)** and *V. parahaemolyticus* **(D)**. Spearman correlation analysis was performed with thresholds of |*ρ*| ≥0.85 and *padj* < 0.05. Red edges: Positive correlations; green edges: Negative correlations. Node size reflects degree centrality (number of connections); larger nodes indicate higher connectivity.

KEGG pathway enrichment results from metabolomics and transcriptomics were integrated to identify pathways dysregulated at both levels (Figures 7A,C). In *S. aureus*, the shared pathways were Histidine metabolism and Pyrimidine metabolism. In *V. parahaemolyticus*, the shared pathways were Glyoxylate and dicarboxylate metabolism, and Valine, leucine, and isoleucine (BCAA) degradation.

Histidine metabolism: The precursor L-histidine was significantly depleted, while downstream intermediates—including glutamic acid, imidazolelactic acid, and ergothioneine—were markedly upregulated. At the transcriptional level, genes encoding histidine biosynthesis (*hisC*, *hisD*), histidine catabolism (*hutI*, *hutG*), and aldehyde dehydrogenase (ALDH) were all differentially expressed (Supplementary Figure S9).

Pyrimidine metabolism: Uracil, thymine, cytidine, and thymidine were significantly upregulated, whereas uridine and 2′-deoxyuridine were markedly downregulated. Genes encoding *de novo* synthesis enzymes (*pyrB*, *pyrC*, *carA*, *pyrG*), salvage pathway enzymes (*upp*, *tdk*), and nucleotide interconversion factors (*ndk*, *pyrH*) all exhibited differential expression (Supplementary Figure S10).

Glyoxylate and dicarboxylate metabolism: TCA cycle intermediates—including citrate, pyruvate, and glycerate—were significantly suppressed, while glutamine and glutamate were upregulated. At the transcriptional level, genes encoding glutamine synthetase (*glnA*), formate dehydrogenases (*fdh3B*, *fdoG*/*fdoH*/*fdoI*), acetyl-CoA acetyltransferase (*atoB*), and serine hydroxymethyltransferase (*glyA*) all showed differential expression (Supplementary Figure S11).

Valine, leucine, and isoleucine (BCAA) degradation: BCAA substrates (L-leucine, L-valine) accumulated, while catabolic intermediates (acetoacetate, 3-methyl-2-oxovalerate) were significantly depleted. Genes encoding the branched-chain *α*-keto acid dehydrogenase (BCKDH) complex (*BCKDHA*, *BCKDHB*, *DBT*), downstream catabolic enzymes (*IVD*, *MCCC1*, *MCCC2*), and acetyl-CoA salvage enzymes (*AACS*, *acsA*, *atoB*) all exhibited differential expression (Supplementary Figure S12).

#### 3.6.2 Gene–metabolite correlation networks

Spearman correlation analysis (|*ρ*| ≥ 0.85, padj < 0.05) was performed to construct gene–metabolite networks within the convergent pathways (Figures 7B,D).

The *S. aureus* network (Figure 7B), encompassing pyrimidine and histidine metabolism, integrated 17 DEGs and 15 DMs with 85 significant edges (82 negative, 3 positive). In pyrimidine metabolism, pyrimidine bases (uracil, thymine) and nucleosides (cytidine, thymidine) showed strong inverse correlations with de novo synthesis genes (*pyrB*, *pyrC*, *pyrG*, *pyrH*, *ndk*) (*ρ* = −0.84 to −0.92), confirming that transcriptional repression of pyrimidine synthesis, rather than enhanced catabolism or transport, drives metabolite accumulation. In histidine metabolism, L-histidine correlated negatively with *hisG* (*ρ* = −0.839), while downstream intermediates (imidazolelactic acid, urocanic acid, imidazoleacetic acid) also exhibited strong negative correlations with *hisG* and *pyrC* (ρ = −0.80 to −0.86).

Network topology analysis identified *pyrC* (encoding dihydroorotase) as the top hub gene (node degree = 11, betweenness centrality = 0.42), exhibiting strong negative correlations with 11 metabolites across both pathways, including uracil (*ρ* = −0.916), thymine (*ρ* = −0.873), L-histidine (*ρ* = −0.951), cytidine (*ρ* = −0.892), thymidine (*ρ* = −0.937), glutamine (*ρ* = −0.845), and glutamate (*ρ* = −0.812). Additional high-degree hubs included *pyrG* (node degree = 9), *pyrB* (node degree = 8), and *hisG* (node degree = 8).

Only three statistically robust positive correlations were observed, all involving *SAOUHSC_02363* and *SAOUHSC_02610*. These genes showed positive correlations with uracil, uridine, thymidine, and a leucine catabolite (*ρ* = 0.81–0.85), suggesting a salvage response to impaired BCAA catabolism.

The *V. parahaemolyticus* network (Figure 7D), encompassing Glyoxylate and dicarboxylate metabolism pathway and BCAA degradation, integrated 22 DEGs and 12 DMs with 143 significant edges (116 negative, 27 positive). In the Glyoxylate and dicarboxylate metabolism, acetyl-CoA and citrate showed strong inverse correlations with *fdh3B* and *VP_RS18185* (*ρ* = −0.888). In the BCAA degradation, L-valine and L-leucine correlated positively with *glnA* and *purU* (*r* > 0.6), but negatively with *fdh3B* and *VP_RS19025* (*ρ* = −0.87 to −0.95). Notably, the catabolic intermediates 3-methyl-2-oxopentanoate and acetoacetate showed exceptionally strong inverse correlations with *fdh3B* (*ρ* = −0.979 and −0.916, respectively).

Network topology analysis identified *fdh3B* (formate dehydrogenase) as the top hub gene (node degree = 11), exhibiting strong negative correlations with 11 metabolites, including L-glutamine, L-valine, L-leucine, glutamate, citrate, acetyl-CoA, 3-methyl-2-oxopentanoate, and acetoacetate. Additional hubs included *glnA* (node degree = 10) and *purU* (node degree = 9). Cross-talk between the two pathways was evident, as acetyl-CoA serves as a shared metabolic hub, and *fdh3B* negatively correlates with metabolites from both pathways, mechanistically linking carbon flux redistribution, nitrogen utilization, and redox homeostasis.

### 3.7 qRT-PCR validation

To confirm the reliability of the RNA-seq data, six selected DEGs were examined by qRT-PCR, as shown in Supplementary Table S7. The expression changes of *pyrC*, *pyrB*, and *hisG* were statistically significant and consistent in direction with the RNA-seq results. The expression changes of *fdh3B* and *glnA* were in the same directions as those detected by RNA-seq; however, the differences measured by qRT-PCR did not reach statistical significance. In contrast, *BCKDHA* was significantly upregulated in the RNA-seq analysis but showed a slight, nonsignificant decrease in the qRT-PCR analysis; therefore, its RNA-seq expression pattern was not validated by qRT-PCR.

The gene-specific divergence between RNA-seq and qRT-PCR may reflect differences in platform normalization and dynamic range, biological variation among the three replicates, variation introduced during reverse transcription, transcript stability or processing, and gene-specific primer amplification efficiency. Therefore, the qRT-PCR results were interpreted as providing partial rather than complete validation of the RNA-seq findings, and the unconfirmed *BCKDHA* result should be interpreted cautiously.

The reference-gene Ct values showed no detectable treatment-associated changes. For *S. aureus*, the mean Ct values of *gyrB* were 16.85 ± 0.07 and 16.89 ± 0.06 in the control and treatment groups, respectively (*p* = 0.463). For *V. parahaemolyticus*, the mean Ct values of *recA* were 11.10 ± 0.54 and 11.05 ± 0.20 in the control and treatment groups, respectively (*p* = 0.885). These results support the absence of a significant treatment-associated shift in the expression of the two reference genes under the present experimental conditions.

## 4 Discussion

The primary contribution of this study lies not in the discovery of a new compound—as cynaroside is a known flavonoid glycoside—but in providing a systematic comparison of its antibacterial mechanisms between Gram-positive (*S. aureus*) and Gram-negative (*V. parahaemolyticus*) bacteria using integrated metabolomic and transcriptomic approaches. This comparative framework suggests that cynaroside induces distinct metabolic responses in the two reference strains examined, potentially reflecting differences in their cell-envelope architectures and physiological characteristics.

### 4.1 Cynaroside treatment is associated with compromised bacterial envelope integrity

Plant-derived compounds have been shown to exert antibacterial effects primarily through multiple mechanisms, including disruption of bacterial cell-membrane integrity, impairment of membrane function, and alteration of membrane permeability (Pérez-Flores et al., 2025; Suh et al., 2025). Cell-envelope damage has frequently been observed following flavonoid treatment; however, such damage may represent either a direct physicochemical effect on envelope components or a secondary consequence of intracellular metabolic and energetic dysfunction.

Cynaroside is a naturally occurring flavonoid glycoside. Consistent with the membrane-associated activities of flavonoids, it has been reported to reduce biofilm formation—a process dependent on bacterial surface and membrane function—in *Pseudomonas aeruginosa* and *Staphylococcus aureus*, and to lower the incidence of mutations leading to ciprofloxacin resistance in *Salmonella typhimurium* (Bouyahya et al., 2023). However, the direct effects of cynaroside on bacterial cell membrane integrity and the associated molecular mechanisms have not been systematically characterized.

In the present study, cynaroside treatment was associated with marked alterations in bacterial morphology, increased electrical conductivity, and elevated extracellular ALP activity in both *S. aureus* and *V. parahaemolyticus*. Collectively, these findings demonstrate that compromised cell-envelope integrity occurs during cynaroside exposure. However, the current experiments do not establish whether envelope damage is the initiating antibacterial event.

It is worth noting that there are significant differences in the responses of the two bacteria to cynaroside. *V. parahaemolyticus* showed a more pronounced increase in electrical conductivity but a lower ALP peak, whereas *S. aureus* showed the opposite pattern. These differences may be related to their distinct cell-envelope architectures. In Gram-negative bacteria, the outer membrane acts as a physical barrier, delaying ALP leakage from the periplasmic space; however, once compromised, it increases susceptibility to external osmotic pressure, resulting in greater electrical conductivity changes (Lundstedt et al., 2020). This phenomenon is consistent with previous reports that flavonoids such as quercetin and kaempferol exert species-specific membrane damage depending on bacterial envelope architecture (Tsuchiya, 2015; Wu et al., 2013). These phenotypic differences provide a foundation for understanding the species-selective action of cynaroside at the molecular level.

It should be noted, however, that while these observations collectively indicate envelope damage, they do not definitively distinguish whether membrane disruption is a primary target of cynaroside or a secondary consequence of metabolic collapse. The observed increases in conductivity and ALP activity could result from either direct membrane perturbation or from the accumulation of toxic metabolites following pathway inhibition. Future studies combining time-kill kinetic assays with early-time-point membrane-permeability measurements, such as PI/SYTO 9 staining within 0.5–2 h of treatment, together with membrane-potential and reactive oxygen species assays, will be required to determine whether envelope disruption precedes or follows broader metabolic dysfunction.

### 4.2 Distinct metabolic reprogramming in *S. aureus* and *V. parahaemolyticus* induced by cynaroside

In this study, conserved metabolic perturbations were observed across multiple core cellular processes. Disruption of amino acid homeostasis (alanine/aspartate/glutamate, cysteine/methionine, and lysine degradation) and nucleotide metabolism (purine and pyrimidine) indicated depletion of precursors essential for protein and nucleic acid synthesis. The uniform enrichment of aminoacyl-tRNA biosynthesis further suggested a translational bottleneck, amplifying global protein synthesis arrest. Concordant enrichment of ABC transporters pointed to impaired nutrient uptake, consistent with the observed membrane permeability increase. These findings align with a recent metabolomic study showing that flavonoid treatment disrupts amino acid metabolism and TCA cycle intermediates in *E. coli* (Chen et al., 2025), supporting that metabolic suppression is a conserved antibacterial mechanism.

Species-specific responses highlight structural and physiological distinctions between the two bacteria. In *V. parahaemolyticus*, metabolite enrichment was most pronounced in the two-component signal transduction system pathway. The two-component system is a core signal transduction mechanism for Gram-negative bacteria to sense environmental changes and make adaptive responses (Omelaniuk et al., 2025). Its enrichment indicates that cynaroside has damaged the environmental sensing and adaptation capabilities of *V. parahaemolyticus*. Concurrently, the observed perturbation of the lipopolysaccharide (LPS) biosynthesis pathway may be associated with altered outer-membrane homeostasis. The SEM and permeability assays independently support the occurrence of cell-envelope damage but do not directly confirm inhibition of LPS biosynthesis or establish the temporal sequence of these events. Regarding energy metabolism, *V. parahaemolyticus* displayed broad-spectrum suppression across central carbon metabolism: glycolysis, TCA cycle, glyoxylate shunt, and anaplerotic reactions were all significantly downregulated. This pattern is consistent with previous reports that plant-derived compounds disrupt the TCA cycle and energy metabolism in bacteria (Jeyaraj et al., 2023). In contrast, *S. aureus* mainly showed interference in the carbon metabolism branch of oxidative phosphorylation, and the disturbance of energy metabolism was relatively limited.

### 4.3 Differential transcriptional regulation in *S. aureus* and *V. parahaemolyticus*

Consistent with recent transcriptomic studies on cynaroside, which demonstrated that it downregulates bacterial virulence genes and activates metabolic stress responses in uropathogenic *E. coli* (Zheng et al., 2026), our analysis revealed significant transcriptional perturbations in both *S. aureus* and *V. parahaemolyticus*.

Both bacteria showed significant perturbations in the expression of genes related to amino acid metabolism, but the specific regulatory patterns differed: *S. aureus* mainly enriched histidine metabolism-related genes, while *V. parahaemolyticus* enriched genes related to BCAA degradation. This pathway has been identified as critical for bacterial survival under stress conditions, and its disruption leads to growth defects and attenuated virulence (Tang et al., 2025). In terms of translational regulation, *S. aureus* mainly showed disturbances in the one-carbon metabolic process, while *V. parahaemolyticus* directly enriched genes related to negative regulation of translation, indicating that the degree of inhibition of protein synthesis at the transcriptional level differed.

Strain-specific transcriptional responses were highly consistent with the metabolomics findings. In *S. aureus*, genes related to metal ion stress response and lipoteichoic acid (LTA) biosynthesis were specifically enriched. LTA biosynthesis has been validated as an effective target for combating *S. aureus*, with small-molecule inhibitors showing potent activity against both growth and biofilm formation (Naclerio et al., 2019). These transcriptional responses are consistent with perturbation of cell-wall homeostasis and metal-ion regulation in *S. aureus*, although they do not demonstrate direct molecular targeting of the cell wall by cynaroside. At the pathway level, *S. aureus* also showed enrichment of teichoic acid biosynthesis and lipid metabolism pathways (phosphatidylinositol signaling, glycerophospholipid metabolism). In *V. parahaemolyticus*, genes related to amino acid-betaine transport, glycine-betaine transport, secretion regulation, and electron transport chain function were enriched, suggesting widespread effects on its transmembrane transport, secretion, and energy metabolism. At the pathway level, *V. parahaemolyticus* specifically enriched the lipopolysaccharide (LPS) biosynthesis pathway. Flavonoids have been shown to inhibit LPS biosynthesis by targeting key enzymes in the heptose biosynthesis pathway (Kim et al., 2021), aligning with our observation that cynaroside compromises the Gram-negative outer membrane barrier.

### 4.4 Integrated multi-omics reveals gram-positive versus gram-negative antibacterial mechanisms of cynaroside

Pyrimidine nucleotides are essential precursors for DNA and RNA synthesis. The observed downregulation of *de novo* pyrimidine biosynthesis genes (*pyrB*, *pyrC*, *pyrG*) and the accumulation of pyrimidine bases (uracil, thymine) suggest that de novo synthesis is blocked, while salvage pathways may be attempting to compensate. This metabolic bottleneck would limit the availability of nucleotide triphosphates required for DNA replication and transcription, ultimately impairing cell division. The strong negative correlation between *pyrC* expression and uracil/thymine levels (*ρ* = −0.916 and −0.873, respectively) further supports this interpretation. The pyrimidine biosynthesis pathway has been recognized as a validated target for antibacterial drug development, and dihydroorotase (*PyrC*) is structurally and mechanistically distinct from its mammalian counterpart, making it an attractive selective target (Lipowska et al., 2019; Truong et al., 2013).

Histidine serves as the precursor for ergothioneine, a potent antioxidant that protects bacteria against oxidative damage. The accumulation of ergothioneine and other downstream metabolites (imidazolelactic acid, urocanic acid) in response to cynaroside treatment suggests a compensatory antioxidant response. This is consistent with the hypothesis that cynaroside induces oxidative stress, triggering a metabolic shift toward antioxidant production at the expense of growth-related pathways. The differential expression of histidine biosynthesis (*hisC*, *hisD*) and catabolism (*hutI*, *hutG*) genes further indicates a re-routing of metabolic flux to prioritize antioxidant defense over biomass production.

The glyoxylate shunt is a critical anaplerotic pathway that allows bacteria to utilize two-carbon compounds (e.g., acetate, fatty acids) for biosynthesis when the tricarboxylic acid (TCA) cycle is impaired. This pathway is particularly important for Gram-negative bacteria, which rely on efficient carbon flux redistribution under stress conditions. Its suppression would reduce the supply of TCA cycle intermediates, limiting both energy production (ATP) and biosynthesis of amino acids and nucleotides. The strong negative correlation between *fdh3B* expression and citrate/acetyl-CoA levels (*ρ* = −0.888) indicates that formate dehydrogenase may be a key regulator of carbon flux redistribution. Formate dehydrogenase (FDH) plays a critical role in bacterial energy metabolism by mediating electron transfer between formate and NAD(P)H; inhibition of FDH function disrupts carbon flux and ATP generation (Yang et al., 2022).

Branched-chain amino acids (valine, leucine, isoleucine) serve as alternative energy sources under stress conditions. The accumulation of BCAAs and depletion of their catabolic intermediates (acetoacetate, 3-methyl-2-oxovalerate) indicate a blockade at the branched-chain *α*-keto acid dehydrogenase (BCKDH) complex step. This would limit acetyl-CoA production from BCAA catabolism, further exacerbating the energy deficit caused by glyoxylate cycle inhibition. The compensatory upregulation of acetyl-CoA salvage genes (AACS, acsA, atoB) suggests an attempt to maintain energy homeostasis despite the blockade. The BCAA degradation pathway has been identified as critical for bacterial survival under stress conditions, and its disruption leads to growth defects and attenuated virulence (Tang et al., 2025).

In terms of *S. aureus* (Gram-positive). The shared pathways between metabolomics and transcriptomics were histidine metabolism and pyrimidine metabolism. In the histidine pathway, L-histidine depletion coupled with accumulation of downstream intermediates (glutamic acid, imidazolelactic acid, ergothioneine) and differential expression of biosynthesis (*hisC*, *hisD*) and catabolism (*hutI*, *hutG*) genes indicated metabolic flux rerouting. Ergothioneine, a potent ROS scavenger synthesized exclusively from L-histidine, accumulated as a compensatory antioxidant response to cynaroside-induced oxidative damage (Zheng et al., 2026). In the pyrimidine pathway, upregulation of uracil, thymine, cytidine, and thymidine alongside differential expression of *de novo* synthesis genes (*pyrB*, *pyrC*, *carA*, *pyrG*) and salvage enzymes (upp, tdk) suggested a metabolic shift toward nucleotide precursor accumulation, likely representing an attempt to sustain translational and replicative capacity under stress. The pyrimidine biosynthesis pathway has been recognized as a validated target for antibacterial drug development (Lipowska et al., 2019). Specifically, dihydroorotase (PyrC), the third enzyme in this pathway, is structurally and mechanistically distinct from its mammalian counterpart and has been confirmed to be essential for *S. aureus* survival, making it an attractive antibacterial drug target (Truong et al., 2013).

Network topology analysis identified *pyrC* as the top hub gene (node degree = 11), exhibiting strong negative correlations with 11 metabolites across pyrimidine and histidine metabolism. This cross-pathway connectivity positions *pyrC* not merely as a pathway-specific enzyme but as a master transcriptional regulator coordinating nitrogen and carbon precursor homeostasis under cynaroside stress. Additional hubs (*pyrG*, *pyrB*, *hisG*) form a core transcriptional module that synchronously represses nitrogen-rich precursor synthesis to conserve energy and redirect resources toward antioxidant defense.

In terms of *V. parahaemolyticus* (Gram-negative). The shared pathways were Glyoxylate and dicarboxylate metabolism and BCAA degradation. In the Glyoxylate and dicarboxylate metabolism, suppression of TCA cycle intermediates (citrate, pyruvate, glycerate) coupled with upregulation of glutamine and glutamate indicated carbon flux disruption. In BCAA degradation, accumulation of L-leucine and L-valine alongside depletion of catabolic intermediates (acetoacetate, 3-methyl-2-oxovalerate) and differential expression of BCKDH complex genes (*BCKDHA*, *BCKDHB*, *DBT*) indicated a blockade at the BCKDH step, resulting in compensatory transcriptional induction of acetyl-CoA salvage pathways.

Network topology analysis identified *fdh3B* (encoding formate dehydrogenase, FDH) as the top hub gene (node degree = 11), exhibiting strong negative correlations with 11 metabolites, including TCA cycle intermediates (citrate, acetyl-CoA) and BCAA metabolites. FDH plays a critical role in bacterial energy metabolism by mediating electron transfer between formate and NAD(P)H; inhibition of FDH function disrupts carbon flux and ATP generation (Yang et al., 2022). Acetyl-CoA serves as a central metabolic hub shared by both pathways, displaying robust inverse correlations with metabolites from each route. FDH acts as a pivotal regulatory nexus, mechanistically linking carbon flux redistribution, nitrogen utilization, and redox homeostasis.

To contextualize our findings within the existing body of knowledge on cynaroside’s antibacterial activity, our study bridges this gap by providing an integrated metabolomic and transcriptomic analysis of cynaroside’s antibacterial mechanisms against both *S. aureus* (Gram-positive) and *V. parahaemolyticus* (Gram-negative). While previous reports have highlighted membrane disruption and ROS induction as key mechanisms, our integrated network analysis further indicates distinct responses in the two reference strains examined: cynaroside treatment was associated with perturbations in pyrimidine and histidine metabolism and cell-wall-related pathways in *S. aureus*, whereas changes in glyoxylate and dicarboxylate metabolism, BCAA degradation, and outer-envelope-related pathways predominated in *V. parahaemolyticus*.

We acknowledge several limitations of this study. First, while our integrated omics analyses reveal associations between cynaroside treatment and metabolic pathway perturbations, they do not demonstrate direct molecular targeting. In addition, although the SEM, conductivity, and ALP-release assays demonstrate treatment-associated cell-envelope damage, the present experimental design cannot determine whether this damage represents an initiating event or a secondary consequence of metabolic, energetic, or redox dysfunction. Resolving this sequence will require time-kill kinetic assays coupled with early-time-point membrane-permeability measurements. Future biochemical studies are needed to establish direct target engagement. Second, the antibacterial activity of cynaroside is moderate compared with conventional antibiotics such as ampicillin. Our primary contribution lies in the mechanistic insights derived from multi-omics analysis, which reveal species-specific metabolic vulnerabilities, rather than in positioning cynaroside as an immediate clinical candidate. Third, the qRT-PCR analysis provided partial rather than complete support for the RNA-seq results. Although *pyrC*, *pyrB*, and *hisG* showed significant and concordant expression changes, *fdh3B* and *glnA* showed only nonsignificant directional agreement, and the RNA-seq upregulation of *BCKDHA* was not reproduced by qRT-PCR. These gene-specific differences may be related to platform normalization, biological variation, reverse-transcription variability, transcript stability, or primer amplification efficiency. Therefore, conclusions regarding the unconfirmed genes, particularly *BCKDHA*, should be interpreted cautiously. Gene-knockout, complementation, and protein-level studies will be required to establish the causal roles of the candidate hub genes. Fourth, only one reference strain of each bacterial species was examined. Therefore, the MIC values and multi-omics responses observed in the present study cannot be directly extrapolated to genetically diverse clinical isolates or drug-resistant phenotypes, including methicillin-resistant *S. aureus* (MRSA). Differences in cell-envelope composition, membrane permeability, efflux activity, resistance determinants, stress-response systems, and metabolic states may substantially influence susceptibility to cynaroside. Although the simultaneous perturbation of several cellular pathways suggests that cynaroside may act independently of a single conventional antibiotic target, the present results do not demonstrate activity against resistant strains. Future studies should evaluate MIC, MBC, and time-kill responses in broader panels of clinical and drug-resistant *S. aureus* and *V. parahaemolyticus* isolates.

## 5 Conclusion

In this study, three known compounds—piperyamine A, cynaroside, and gallic acid—were isolated from *T. arguta* leaves. Among them, cynaroside exhibited the most potent antibacterial activity, with MIC values of 31.25 μg/mL against *S. aureus* and 62.5 μg/mL against *V. parahaemolyticus*. Phenotypic experiments confirmed that cynaroside treatment is associated with disruption of bacterial cell wall and membrane integrity, although whether this represents a primary or secondary effect remains to be determined. The primary contribution of this study, however, is the elucidation of species-specific metabolic pathway perturbations induced by cynaroside, as revealed by integrated multi-omics analysis. In *S. aureus* (Gram-positive), cynaroside primarily perturbs pyrimidine and histidine metabolism, with *pyrC* as the central hub gene coordinating nitrogen precursor suppression and cell wall synthesis disruption. In *V. parahaemolyticus* (Gram-negative), cynaroside mainly perturbs glyoxylate and dicarboxylate metabolism and valine, leucine, and isoleucine (BCAA) degradation, with *fdh3B* as the top hub gene disrupting carbon flux and ATP generation, coupled with outer membrane destabilization. Within the two reference strains examined, cynaroside treatment was associated with distinct metabolic and cell-envelope responses, involving pyrimidine and histidine metabolism and cell-wall-associated pathways in *S. aureus*, and glyoxylate and dicarboxylate metabolism, BCAA degradation, and outer-envelope-associated pathways in *V. parahaemolyticus*. These findings suggest that cell-envelope injury and metabolic dysfunction may be interconnected, although their temporal and causal relationships remain unresolved. The extent to which these responses apply to clinical or drug-resistant isolates requires further investigation.

## Data Availability

The original contributions presented in the study are publicly available. This data can be found here: https://www.ncbi.nlm.nih.gov/bioproject/PRJNA1515445.
