# Supplementary material for: Cynaroside targets distinct metabolic pathways in gram-positive and gram-negative pathogenic bacteria: an integrated multi-omics study
Source: Front Microbiol. 2026 Aug 26;17:1888286. doi: 10.3389/fmicb.2026.1888286 (PMC13553902; doi:10.3389/fmicb.2026.1888286)
Supplement: Supplementary file 1 [file Supplementary_file_1.DOCX]

**Supplementary Figures**

**Supplementary Figure S1** ¹H NMR spectrum (400 MHz, CDCl₃) of compound 1 (Piperyamine A).

**Supplementary Figure S2** ¹³C NMR spectrum (101 MHz, CDCl₃) of compound 1.

**Supplementary Figure S3** ¹H NMR spectrum (600 MHz, DMSO-d₆) of compound 2 (cynaroside).

**Supplementary Figure S4** ¹³C NMR spectrum (151 MHz, DMSO-d₆) of compound 2.

**Supplementary Figure S5** ¹H NMR spectrum (600 MHz, CD₃OD) of compound 3 (gallic acid).

**Supplementary Figure S6** 13C NMR spectrum (150 MHz，CD_3_OD) compound 3 (gallic acid).

**Supplementary Figure S7** Overview of metabolomic profiling of *S. aureus* and *V. parahaemolyticus* following cynaroside treatment.

(**A**) Venn diagram showing the number of detected metabolites in *S. aureus* control (SA-meta-C) and treated (SA-meta-T) groups.

(**B**) Venn diagram showing the number of detected metabolites in *V. parahaemolyticus* control (VP-meta-C) and treated (VP-meta-T) groups.

(**C**) HMDB superclass classification of identified metabolites in *S. aureus*.

(**D**) HMDB superclass classification of identified metabolites in *V. parahaemolyticus*.

**Supplementary Figure S8** Principal component analysis (PCA) of transcriptomic data from *S. aureus* and *V. parahaemolyticus* following cynaroside treatment.

****(A)**** PCA score plot of *S. aureus* samples. Clear separation was observed between the control group (SA-tran-C) and the cynaroside-treated group (SA-tran-T). Each point represents an individual biological replicate (n = 3 per group).

****(B)**** PCA score plot of *V. parahaemolyticus* samples. The control group (VP-tran-C) and treated group (VP-tran-T) showed distinct separation, with good clustering within groups (n = 3 per group).

**Supplementary Figure S9** Histidine metabolism in *S. aureus*

**Supplementary Figure S10** pyrimidine metabolism in *S. aureus*

**Supplementary Figure S11** Glyoxylate and dicarboxylate metabolism in *V. parahaemolyticus*

**Supplementary Figure S12 Valine, leucine, and isoleucine (BCAA) degradation in V. parahaemolyticus.**

**Supplementary Tables**

**Supplementary Table S1** Primer information in qRT-PCR validation

| Primer name | Primer sequence(5'to3') |
| --- | --- |
| *pyrC*-L | AGGAATTGGGGACTGGGTTT |
| *pyrC*-R | CCTTGAGACATTCGAGCAGC |
| *pyrB*-L | AGGCTAAGGTTGAGGCCAAT |
| *pyrB*-R | GCGGCAACAGCTCAAAATTC |
| *hisG*-L | CCAAATGCACTGTTCGTCCA |
| *hisG*-R | GACAAACACAGCCCAAGACA |
| *fdh3B*-L | ACATTTCATTTCCCCGCCTC |
| *fdh3B*-R | CAGGTTGTTGGCTAAGCAGG |
| *glnA*-L | ATGCGGATCATCAGGCAGTA |
| *glnA*-R | GCTCTTCAAGAACCGGCTTC |
| *BCKDHA*-L | ACTTGCCAGTTGTGTCGTTC |
| *BCKDHA*-R | AGCAAACCCTTCTCTCTGCA |
| *gyrB*-L | ACATTACAGCAGCGTATTAG |
| *gyrB*-R | CTCATAGTGATAGGAGTCTTCT |
| *recA*-L | GCTAGTAGAAAAAGCGGGTG |
| *recA*-R | GCAGGTGCTTCTGGTTGAG |

## **Supplementary Table S2** ¹H and ¹³C NMR data for compound 1 (piperyamine A) in CDCl₃

| **Position** | **δ¹³C (ppm)** | **δ¹H (ppm), multiplicity (J in Hz)** |
| --- | --- | --- |
| 1 | 59.58 | 4.15, d (7.1) |
| 2 | 123.21 | 5.41, t (7.0) |
| 3 | 140.49 | - |
| 4 | 40.02 | 1.99, t (8.3) |
| 5 | 32.94 | 1.61-1.58, m* |
| 6 | 37.58 | 1.34-1.19, m* |
| 7 | 25.28 | 1.34-1.19, m* |
| 8 | 37.51 | 1.34-1.19, m* |
| 9 | 36.81 | 1.34-1.19, m* |
| 10 | 29.51 | 1.34-1.19, m* |
| 11 | 37.44 | 1.34-1.19, m* |
| 12 | 24.62 | 1.34-1.19, m* |
| 13 | 24.95 | 1.34-1.19, m* |
| 14 | 39.52 | 1.34-1.19, m* |
| 15 | 28.13 | 1.61-1.58, m* |
| 16 | 22.78 | 0.88-0.80, m* |
| 17 | 22.87 | 0.88-0.80, m* |
| 18 | 16.33 | 1.67, s |
| 19 | 32.08 | 1.61-1.58, m* |
| 20 | 19.87 | 0.88-0.80, m* |
| 21 | 19.90 | 0.88-0.80, m* |

## **Supplementary Table S3** ¹H and ¹³C NMR data for compound 2 (cynaroside) in DMSO-d₆

| **Position** | **δ¹³C (ppm)** | **δ¹H (ppm), multiplicity (J in Hz)** |
| --- | --- | --- |
| **Aglycone** |  |  |
| 2 | 164.59 | - |
| 3 | 104.16 | 6.45, s |
| 4 | 182.13 | - |
| 5 | 161.93 | - |
| 6 | 99.29 | 6.20, d (2.2) |
| 7 | 164.35 | - |
| 8 | 94.31 | 6.68, d (2.2) |
| 9 | 157.75 | - |
| 10 | 103.33 | - |
| 1' | 121.96 | - |
| 2' | 113.82 | 7.40, d (2.3) |
| 3' | 146.20 | - |
| 4' | 150.16 | - |
| 5' | 116.48 | 6.90, d (8.3) |
| 6' | 119.46 | 7.44, dd (2.3, 8.3) |
| **Glucose** |  |  |
| 1" | 70.87 | - |
| 2" | 77.57 | - |
| 3" | 67.41 | - |
| 4" | 76.40 | - |
| 5" | 51.67 | - |
| 6" | - | - |

**Phenolic OH**: δ 12.98 (s, 5-OH), 10.82 (s, 7-OH), 9.91 (s, 4'-OH), 9.50 (s, 3'-OH)

**Supplementary Table S4** ¹H and ¹³C NMR data for compound 3 (gallic acid) in CD₃OD

| **Position** | **δ¹³C (ppm)** | **δ¹H (ppm), multiplicity (J in Hz)** |
| --- | --- | --- |
| COOH | 170.26 | - |
| 1 | 120.89 | - |
| 2 | 109.94 | 6.98, s |
| 3 | 144.38 | - |
| 4 | 137.94 | - |
| 5 | 144.38 | - |
| 6 | 109.94 | 6.98, s |

Note: Positions 2 and 6 are equivalent; positions 3 and 5 are equivalent.

**Supplementary Table S5** Minimum inhibitory concentration (MIC) of compounds 1–3 against S. aureus and V. parahaemolyticus

| **Compounds** | **MIC against S. aureus (µg/mL)** | **MIC against V. parahaemolyticus (µg/mL)** |
| --- | --- | --- |
| Piperyamine A (compound 1) | 500 | 500 |
| cynaroside (compound 2) | 32.15 | 62.5 |
| Gallic acid (compound 3) | 62.5 | 125 |
| Ampicillin (positive control) | 2 | 4 |

**Supplementary Table S6** Quality control metrics of RNA sequencing data for *S. aureus* and *V. parahaemolyticus*.

| **Sample Name** | **Raw reads** | **Clean Reads** | **Clean Q30**  **(%)** | **Uniquely Mapped Reads Ratio**  **(%)** |
| --- | --- | --- | --- | --- |
| SA-tran-C-1 | 20934066 | 20851366 | 97.1 | 91.09 |
| SA-tran-C-2 | 20976060 | 20897840 | 96.93 | 94.82 |
| SA-tran-C-3 | 23776116 | 23623574 | 96.83 | 94.1 |
| SA-tran-T-1 | 24951700 | 24850980 | 97.13 | 94.79 |
| SA-tran-T-2 | 22205568 | 22118618 | 95.55 | 95.66 |
| SA-tran-T-3 | 21050266 | 20971770 | 96.99 | 95.84 |
| VP-tran-C-1 | 26084824 | 25984078 | 96.81 | 94.6 |
| VP-tran-C-2 | 25218148 | 25126392 | 96.87 | 94.65 |
| VP-tran-C-3 | 24408282 | 24319376 | 96.77 | 94.66 |
| VP-tran-T-1 | 24693338 | 24578170 | 96.81 | 94.2 |
| VP-tran-T-2 | 27011528 | 26885266 | 96.82 | 94.66 |
| VP-tran-T-3 | 22760616 | 22665946 | 96.79 | 94.47 |

**Supplementary Table S7** Comparison of qRT-PCR and RNA-seq results

| **Gene** | **RNA-seq log₂FC** | **RNA-seq adjusted P value** | **qRT-PCR fold change (mean ± SD)** | **qRT-PCR P value** | **Validation outcome** |
| --- | --- | --- | --- | --- | --- |
| *pyrC* | 1.3083 | 5.6508 × 10⁻⁵ | 5.39 ± 0.26 | <0.001 | Consistent |
| *pyrB* | 1.6984 | 1.5573 × 10⁻⁶ | 9.22 ± 0.48 | <0.001 | Consistent |
| *hisG* | 1.9604 | 1.4092 × 10⁻¹² | 0.44 ± 0.02 | <0.001 | Consistent |
| *fdh3B* | 2.1066 | 1.8377 × 10⁻¹⁹ | 1.17 ± 0.12 | 0.480 | Consistent trend (NS) |
| *glnA* | −1.0094 | 9.9501 × 10⁻¹⁸ | 0.59 ± 0.10 | 0.093 | Consistent trend (NS) |
| *BCKDHA* | 1.8266 | 1.7900 × 10⁻³⁰ | 0.91 ± 0.15 | 0.651 | Not validated |
